# Transcriptomic Analysis Reveals the Inhibitory Mechanism of Fisetin Against the Pathogenicity of *Aeromonas hydrophila*

**DOI:** 10.3390/ani15162415

**Published:** 2025-08-18

**Authors:** Jing Dong, Xinwei Ma, Shengping Li, Shun Zhou, Qiuhong Yang, Xiaohui Ai

**Affiliations:** 1 Yangtze River Fisheries Research Institute, Chinese Academy of Fishery Sciences, Wuhan 430223, China; 2 College of Veterinary Medicine, Hunan Agricultural University, Changsha 410125, China; 3 College of Fisheries and Life Science, Shanghai Ocean University, Shanghai 201306, China

**Keywords:** *Aeromonas hydrophila*, RNA-seq, anti-virulence, fisetin, aquaculture

## Abstract

*Aeromonas hydrophila* is an important zoonotic bacterium that is related to multiple diseases in humans, terrestrial animals, and aquatic animals. Bacterial septicemia caused by *A. hydrophila* often results in high mortality and severe economic losses. Antibiotics, the major approach to dealing with bacterial infections, are limited due to the occurrence of antibiotic resistance. Anti-virulence strategies provide a promising approach to combat resistant bacterial infections. Here, growth curves, hemolysis, biofilm formation, and animal studies were performed to investigate the effect of fisetin against *A. hydrophila.* Moreover, RNA-seq technology was employed to determine the potent mechanism of fisetin. The results showed that fisetin could dose-dependently reduce the hemolytic activities mediated by aerolysin and hinder biofilm formation. Animal studies showed that treatment with 50 mg/kg fisetin could remarkably reduce the mortality to 40% in the infected group compared with fish in the fisetin-free group. Further, transcriptome analysis demonstrated that there were 565 differentially expressed genes (DEGs) after treatment with 16 μg/mL fisetin. Fisetin significantly impacted the pathways related to oxidative phosphorylation, the citrate cycle, and virulence factor regulation. Furthermore, 159 virulence-related genes were influenced after fisetin treatment. Collectively, these findings revealed that fisetin could mitigate the pathogenicity of *A. hydrophila* by affecting oxidative phosphorylation and the citrate cycle pathway as well as inhibiting the production of virulence factors. The study not only identified a powerful substance for managing *A. hydrophila*-associated diseases in aquaculture but also clarified the mechanism of plant medicines in controlling diseases caused by bacterial pathogens.

## Simple Summary

Freshwater aquaculture plays a major role in meeting the increasing demands for high-quality proteins in the form of food fish. However, the spread of bacterial diseases is threatening the sustainable growth of the industry. Novel drug alternatives to antibiotics are urgently needed due to the emergence of antibiotic resistance. In this study, we determined the protective effect of fisetin against *A. hydrophila* infection in fish by decreasing the virulence factors. Further transcriptome analysis determined the mechanism of fisetin. The study showed that an anti-virulence strategy is a feasible approach for developing anti-infective drugs that combat *A. hydrophila*.

## 1. Introduction

Aquaculture has become the fastest-growing sector among the food production system to find a solution to the increasing demands of high-quality proteins and nutritional deficiencies globally [1,2]. The average consumption of aquatic foods from marine and freshwater environments per capita has increased to 20.21 kg per year, and developing and small-island countries rely more on aquatic products [2]. Therefore, aquaculture is crucial for ensuring food security. However, bacterial diseases break out every year, not only resulting in heavy economic losses to the industry but also threatening animal welfare after the intensive development of aquaculture [3]. Over 13 bacterial genera are responsible for bacterial infections in aquaculture all over the world [3]. It is known that there are over 200 fish diseases in cultured fish in China, and bacterial infections can cause about 15–20% loss of total production [4]. Antibiotics are regarded as the leading solutions for treating bacterial diseases and are frequently used in aquaculture.

*Aeromonas hydrophila*, a ubiquitous bacterium distributed in aquatic environments, causes a number of fish diseases, including septicemia, red sore disease, and skin ulcers, which often lead to high mortality in aquaculture [5]. Moreover, the bacteria distributed in water and aquatic foods can be transmitted to humans and cause a variety of clinical disease manifestations in humans from mild soft tissue infection to life-threatening septicemia [6]. Since the first attempt of sulfonamide in dealing with furunculosis in trout, a number of antibiotics have been introduced to aquaculture and have become the preferred approach in combating bacterial diseases in aquaculture [7,8]. However, resistance to multiple classes of antibiotics was observed in *A. hydrophila* strains, limiting the treatment options in aquaculture [9]. Therefore, it is important to develop novel approaches to controlling *A. hydrophila* infections to ensure the high-quality development of the aquaculture industry and human health. Virulence factors produced by *A. hydrophila*, such as hemolysin, aerolysin, cytosine, gelatinase, enterotoxin, and biofilm, help the bacteria establish and maintain the infections [10,11]. Consequently, screening drugs targeting the activities or productions of virulence factors might be a useful way of combating drug-resistant *A. hydrophila* infections in aquaculture.

Herbal medicines and their major chemical compounds with anti-bacterial, growth-promoting, and anti-inflammatory effects are widely used in aquaculture in China and have been regarded as ideal alternatives to antibiotics in dealing with bacterial infections [12,13]. Fisetin, belonging to the flavonoids, is the chemical component of several vegetables and fruits, including strawberries, apples, nuts, and grapes [14,15]. Fisetin exhibits several biological functions, including anti-oxidation, anti-inflammatory, and anti-cancer effects [16,17,18]. Nevertheless, the inhibitory effect of fisetin against bacterial pathogens isolated from aquaculture was relatively unknown. This study was performed to elucidate the mode of action of fisetin against the pathogenicity of *A. hydrophila* and provide a novel agent to manage *A. hydrophila*-associated diseases in aquaculture.

## 2. Materials and Methods

### 2.1. Bacterial Strain and Reagents

*A. hydrophila* XS-91-4-1 is a clinical isolate stored in our laboratory. Fisetin was obtained from Sichuan Vicky Biotechnology Co., Ltd. (Chengdu, China). For in vitro studies, fisetin was dissolved in DMSO at the concentration of 40,960 μg/mL. For animal studies, fisetin was prepared in PBS, and the pH was adjusted to 9.0.

### 2.2. Minimal Inhibitory Concentration (MIC) Determination

The MIC assay was conducted to assess the susceptibility of fisetin using the micro-dilution method in a 96-well cell plate in accordance with CLSI [19]. Fisetin was dissolved in 100 μL MHB medium in each well to obtain final concentrations ranging from 512 to 1 μg/mL after a 2-fold serial dilution. An overnight bacterial suspension was sub-cultured in LB medium at 28 °C to the mid-logarithmic phase, and then bacterial cells were harvested by centrifugation. The concentration of cells was adjusted to 1 × 10^6^ cfu/mL by MHB after washing with PBS. Then, 100 μL of bacterial cells was mixed with the same volume of fisetin in a 96-well plate. MHB with bacterial cells was employed as the fisetin-free control, while MHB medium only served as the negative control. After an incubation at 28 °C for 16–18 h, the MIC was defined as the lowest concentration without visible growth of bacteria.

### 2.3. Growth Curve Assay

Growth curve assays were carried out to examine the effect of adding fisetin on *A. hydrophila* XS-91-4-1 growth after 5 h. The bacteria after being cultured overnight were sub-inoculated into 100 mL of fresh LB medium at a ratio of 1:100 and further cultured to the early logarithmic phase, which was ascertained by measuring the optical density at 600 nm (OD_600nm_) and obtaining a value of 0.3. The inoculum was divided into 5 conical flasks; each flask contained 10 mL bacterial suspension. Then, fisetin at final concentrations of 2, 4, 8 and 16 μg/mL was mixed with the suspension, while bacterial culture supplemented with DMSO was defined as the fisetin-free control. The bacterial suspensions in each flask were cultured in a thermostatic shaker at 28 °C for 5 h, and the growth trends were determined by measuring OD_600nm_.

### 2.4. Hemolytic Activity Assay

*A. hydrophila* XS-91-4-1 in the early logarithmic phase (OD_600nm_ = 0.3) was equally separated into 5 flasks and fisetin was added to make final concentrations of 0, 2, 4, 8 and 16 μg/mL. The inoculates were cultured to an OD_600nm_ of 1.5 and then were centrifuged (6000 g, 4 °C) to obtain cell-free supernatants. Bacterial supernatants were used for determining the influence of fisetin on hemolysis of supernatants after activation with trypsin. The hemolytic activity determination reaction was placed in 1.5 mL tubes and included sheep erythrocytes, bacterial supernatants, and hemolytic buffer at volumes of 25, 100, and 875 μL, respectively. After gentle mixing, the tubes were incubated at 37 °C for 15 min. The tubes were then centrifuged to remove the unlysed blood cells, and the OD_543nm_ was determined to calculate the hemolytic activities after fisetin treatment. Supernatants collected from bacteria co-cultured without fisetin treatment served as 100% hemolysis.

### 2.5. Western Blot

The contents of aerolysin in bacterial supernatants obtained as described above were measured using a western blot assay. First, the BCA method was employed to measure the content of proteins in the samples. Then, electrophoresis was performed using a SurePAGE Bis-Tris gel after sampling with loading buffer. The gel containing aerolysin was transferred to a PVDF membrane using a wet transfer cell. The membrane containing target proteins was then incubated with skim milk to block the extra sites for 2 h. Following the incubation with a primary anti-aerolysin antibody for 1 h, the membrane was then incubated with an HRP-conjugated goat anti-rabbit antiserum for 1 h. ECL detection reagents were used and co-incubated with the membrane. Proteins were detected, and images were captured by a chemiluminescence imaging system.

### 2.6. Biofilm Formation Assay

The impact of fisetin on biofilm production was evaluated using the crystal violet staining method in a 96-well plate [20]. Fisetin was diluted 2-fold in the plate at volumes of 100 μL. Then, the same volumes of bacterial suspension were added to produce fisetin concentrations of 0, 2, 4, 8 and 16 μg/mL in each well. The plate was static cultured at 37 °C in a humidity incubator for 24 h. The values of OD_600nm_ were measured using a microplate reader to confirm the bacteria in all wells were in the stationary phase. Bacterial cells in the planktonic state were discarded by washing with PBS, and the plate was air-dried to fix the bacterial cells. Then, 0.5% crystal violet was equally added to each well to stain the bacterial cells in the plate. After washing, crystal violet was released by the addition of acetic acid. The biofilm after an incubation with fisetin was evaluated by determining the OD_570nm_.

### 2.7. Animal Study

The therapeutic effect of fisetin alleviating *A. hydrophila* infection was determined using healthy channel catfish obtained from our breeding center. Ninety channel catfish (weighing 100 ± 10 g, born in the same year) were randomly separated into 3 treatment groups, and each treatment group contained triplicates of 10 fish per tank. Tanks containing 500 L water were used to maintain the fish for 7 days before use. The bacterial strain was cultured to an OD_600nm_ of 1.0, and bacterial cells were collected by centrifugation. After washing 3 times with PBS, the density was adjusted to 1.5 × 10^9^ cfu/mL using McFarland standards [21]. After being anaesthetized by MS-222 at 40 g/m^3^ for 5 min, fish in the fisetin-free control and fisetin-treated groups were challenged with 100 μL bacterial suspension (containing 1.5 × 10^8^ bacterial cells) intraperitoneally, while the same volume of PBS was injected into fish in the negative control group. Then, fish in the fisetin-treated group were orally given 50 mg/kg fisetin 6 h post infection using a gavage needle. The treatment was maintained for 3 days. Fish in the fisetin-free and negative control groups were treated with sterile PBS (pH 9.0). Fish in all groups were observed for 10 days, and mortality was determined.

### 2.8. RNA Sequencing Analysis

Bacterial suspension reached OD_600nm_ of 0.3, and then the incubation was divided into 2 flasks. Here, 16 μg/mL fisetin was then mixed with the bacterial suspension in one of the flasks, while the bacterial suspension in another flask without fisetin was used as the fisetin-free control. Both groups were set up as triplicates. To collect bacterial cells after fisetin treatment, centrifugation (6000 g, 5 min, 4 °C) was performed after the OD_600nm_ values of bacterial suspension reached 1.5. Then, the cells were stored in liquid nitrogen immediately. TRIzol^®^ Reagent was applied to separate the RNA from bacterial cells after fisetin treatment according to the instructions supplied by the manufacturer. The concentration and quality of the RNA were measured using a Nanodrop 2000 and the Agilent 5300 fragment analyzer system. RNA samples with high-quality were used for constructing the sequence library after removing rRNA with a ribo-Zero rRNA Removal Kit. cDNA was prepared by a commercial cDNA synthesis kit, and sequences of the samples were used for whole-transcriptome sequencing (RNA-Seq) by Shanghai Majorbio Bio-pharm Technology Co., Ltd. Data generated from the NovaSeqXPlus platform were then analyzed using the Cloud Platform of Majorbio. SeqPrep (Version 2011) and Sickle (Version 1.33) were used to guarantee the quality of the RNA-seq data. The clean reads were then aligned with the genome of *A. hydrophila* ATCC 7966 using Bowtie 2 (Version 1.2.2). The expression levels were determined based on TPM (transcripts per million reads) values. Gene Ontology (GO) (http://www.geneontology.org/, accessed on 23 October 2024) and Kyoto Encyclopedia of Genes and Genomes (KEGG) (http://www.genome.jp/kegg/, accessed on 23 October 2024) were used to analyze the function annotation and pathway enrichment of the differentially expressed genes (DEGs). Moreover, the virulence factor database (VFDB) (https://www.mgc.ac.cn/VFs/main.htm, accessed on 23 October 2024) was used to determine the impact of fisetin on the expression of virulence factors.

### 2.9. Statistical Analysis

The significance of the hemolysis assay and biofilm formation between fisetin-treated groups and the fisetin-free group was evaluated using the student *t*-test method. Survival rates of the animal study were determined with Kaplan–Meier estimates and log-rank tests. DESeq2 (Version 1.42.0) was employed to determine the DEGs between fisetin-treated and control groups, and statistical significance was defined as data with a fold change > 2 and adjusted *p* < 0.05.

## 3. Results

### 3.1. Impact of Fisetin on Bacterial Growth

**Figure 1:**
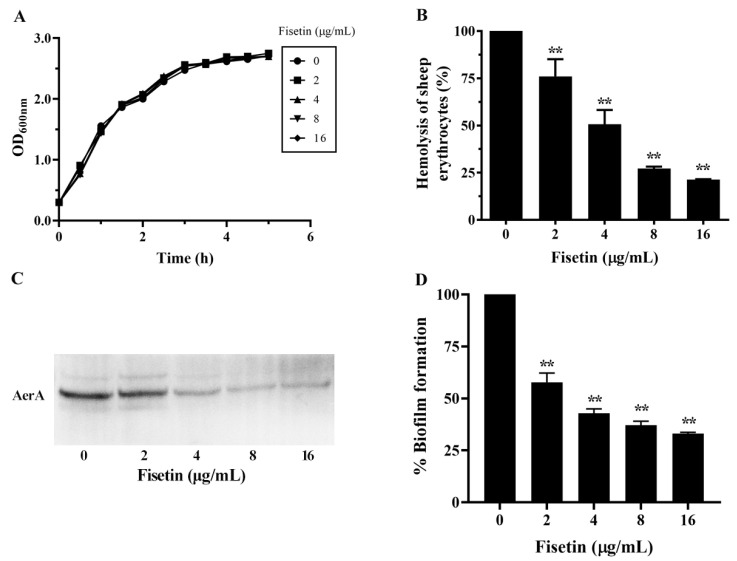
Fisetin reduced the hemolysis and biofilm formation at concentrations ranging from 2 to 16 μg/mL. (**A**), Growth curves of *A. hydrophila* XS-91-4-1 co-cultured with fisetin; (**B**), hemolytic activities of bacterial supernatants after co-incubation with fisetin; (**C**), the relative quantity of aerolysin in bacterial supernatants co-cultured with fisetin; (**D**), fisetin inhibited the biofilm formation of *A. hydrophila*. Data shown in Figure 1A are the mean of three independent assays, while data in Figure 1B,D are the mean ± SD of three independent assays. Data in Figure 1A,B,D are analyzed using the student *t*-test, ** indicates *p* < 0.01.

The MIC of fisetin against *A. hydrophila* XS-91-4-1 was 128 μg/mL, suggesting that fisetin had only minimal bacteriostatic effect. The growth curve assay showed that the tested bacterial strain co-cultured with fisetin from 2 to 16 μg/mL had similar growth trends as the fisetin-free group (Figure 1A), indicating that fisetin had no role in affecting the growth of *A. hydrophila* under the experimental conditions described above.

### 3.2. Influence of Fisetin on the Hemolytic Activities of Bacterial Supernatants

Aerolysin can be secreted into bacterial supernatant and results in hemolysis. Thus, the hemolysis assay was conducted to measure the inhibition of hemolysis mediated by aerolysin after co-culture with fisetin. Fisetin could decrease the hemolysis induced by aerolysin after co-incubation with fisetin at certain concentrations (Figure 1B). The hemolytic activity declined to 76.03 ± 9.13%, 50.70 ± 7.52%, 27.18 ± 1.05%, and 21.34 ± 0.25% with fisetin at 2, 4, 8 and 16 μg/mL compared with the fisetin-free group (Figure 1B). Statistical significance was observed when fisetin reached 2 μg/mL and above. Then, western blot results showed that the quantity of aerolysin also declined following the increasing concentrations of fisetin in bacterial supernatants (Figure 1C). When fisetin reached 8 and 16 μg/mL, aerolysin could hardly be detected in the supernatants. The outcomes suggested that fisetin could reduce aerolysin-induced hemolytic activity by hindering the secretion of aerolysin.

### 3.3. Impact of Fisetin on Biofilm Production of A. hydrophila

Biofilm formed by *A. hydrophila* can regulate the physiological and metabolic functions and result in the high resistance to antibiotics and sanitizers [22]. Therefore, biofilm is identified as a virulence factor related to iterative and continual infections. As shown in Figure 1D, biofilm formation was inhibited after co-incubation with fisetin. The biofilm decreased to 57.76 ± 4.42%, 42.81 ± 2.18%, 37.09 ± 1.95% and 33.08 ± 0.62% compared with the fisetin-free group. After treatment with fisetin at 2 to 16 μg/mL, the amount of biofilm was significantly inhibited.

### 3.4. Impact of Fisetin on the Survivability of a Fish Infection Model

**Figure 2:**
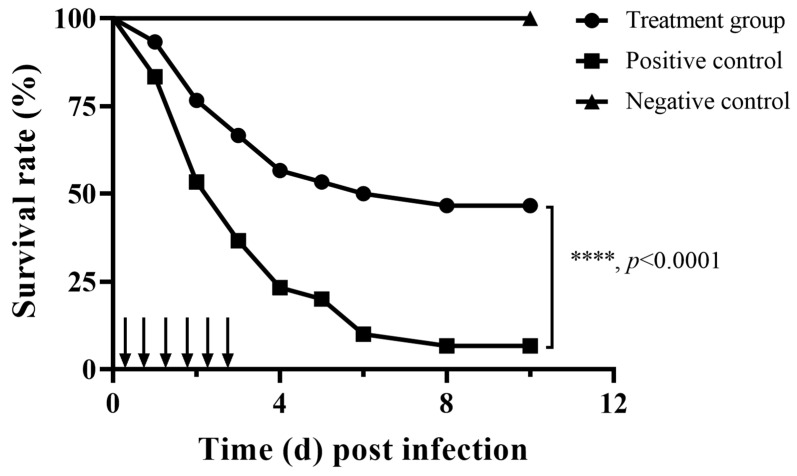
The impact of fisetin on the survival rates of fish challenged with *A. hydrophila*. ↓ indicates the time points when fisetin or PBS was given to fish in each group. Each group contained triplicates of 10 fish. ****: The significance was determined using Kaplan–Meier estimates and log-rank tests (*p* < 0.0001).

Aerolysin could determine the pathogenicity of *A. hydrophila* [23,24]. Thus, the potent protective effect of fisetin against *A. hydrophila* infection was determined by establishing an infection model. Mortality in the fisetin-free group started within 24 h post infection. The total mortality after 10 days was 93.33%, while the mortality in the fisetin group was 53.33% (Figure 2).

Fish in the negative control group were healthy, and the survival rate of the group was 100%, indicating that deaths in the fisetin-free and fisetin groups were caused by the challenge of *A. hydrophila*. The survivability of the fisetin-treated group was significantly enhanced by the treatment with fisetin, suggesting that fisetin could partly neutralize the pathogenicity of *A. hydrophila* by restraining one or more of the virulence factors.

### 3.5. DEGs Determination

**Figure 3:**
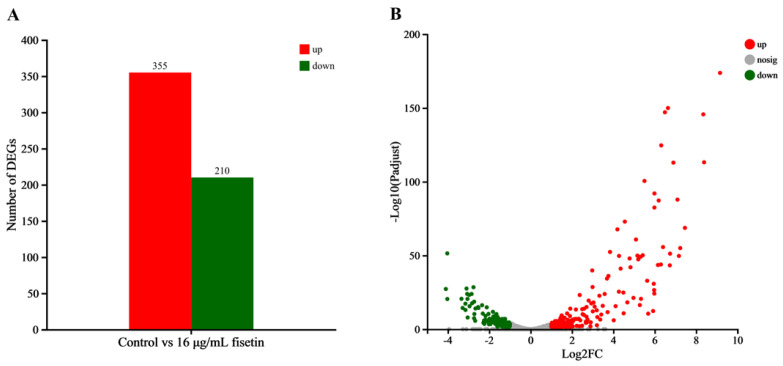
Expression profiles of *A. hydrophila* after treatment with fisetin at 16 μg/mL compared with untreated control. (**A**), Numbers of DEGs, with red bars indicating up-regulated genes, green bars indicating down-regulated genes; (**B**), volcano plot of DEGs in fisetin-treated *A. hydrophila*. Both the 16 μg/mL fisetin-treated and fisetin-free groups were performed in triplicates. The DEGs were analyzed based on |log2(fold change)| > 1 and an adjusted *p*-value < 0.05.

According to the results of RNA-Seq, high-quality clean reads ranged from 2.03 to 2.35 million for the fisetin-free group and the fisetin-treated group with a sequencing error < 0.025%, Q20 > 98%, and Q30 > 96% (Table 1). Moreover, clean reads ranging from 91.95% to 95.53% described above were mapped to the genome of *A. hydrophila* ATCC 7966 after alignment (Table 2). According to the quality control results, data acquired in the present study could satisfy further bioinformatics determination, and data involved were submitted to the NCBI SRA database (Accession No.: PRJNA1237511). In total, 4122 expressed genes were determined in both groups, among which 565 were DEGs with 355 up-regulated genes and 210 down-regulated genes (Figure 3A,B).

### 3.6. GO Function Analysis

**Figure 4:**
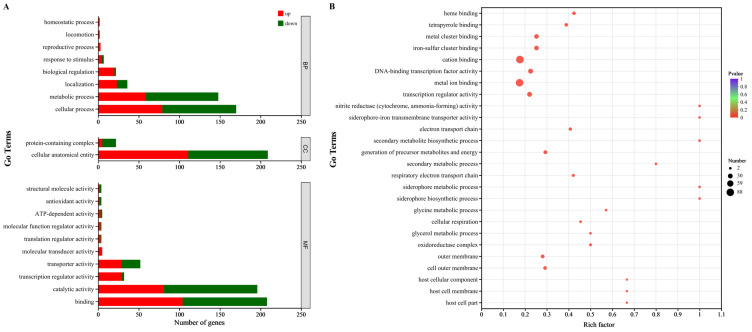
DEGs between the control and fisetin-treated bacterial cells analyzed by GO. (**A**), Function categorization of DEGs, BP indicates biological process, CC indicates cellular component, MF indicates molecular function; (**B**), GO enrichment analysis of DEGs.

A total of 23 GO terms were assigned to 423 DEGs, including 8 biological process terms, 2 cellular component terms and 13 molecular function terms (Figure 4A). In the biological process category, the main distributed terms were cellular process (GO: 0009987, containing 79 up-regulated genes and 91 down-regulated genes), metabolic process (GO:0008152, containing 59 up-regulated genes and 89 down-regulated genes) and localization (GO:0051179, containing 23 up-regulated genes and 13 down-regulated genes). In the cellular component category, cellular anatomical entity (GO:0110165, containing 111 up-regulated genes and 98 down-regulated genes) was the main distributed term. In the molecular function category, the main distributed terms were binding (GO:0005488, containing 104 up-regulated genes and 104 down-regulated genes), catalytic activity (GO:0003824, containing 81 up-regulated genes and 115 down-regulated genes) and transporter activity (GO:0005215, containing 29 up-regulated genes and 23 down-regulated genes).

### 3.7. GO Enrichment Analysis

The result of GO enrichment analysis revealed that fisetin could significantly impact cation binding, metal ion binding, transcription regulator activity, DNA-binding transcription factor activity, etc. (Figure 4B).

### 3.8. KEGG Function Analysis

**Figure 5:**
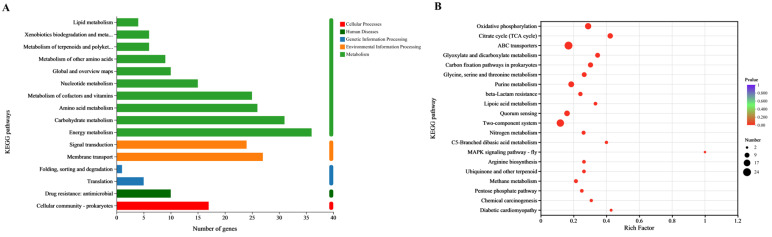
KEGG analysis of DEGs between the control and fisetin-treated bacterial cells. (**A**), KEGG pathway determination of DEGs, (**B**), KEGG enrichment determination of DEGs.

The KEGG pathway classification of DEGs is shown in Figure 5A. Most DEGs were centralized in the categories of metabolism, environmental information processing, cellular processes, human diseases, and genetic information processing, which specifically in energy metabolism, carbohydrate metabolism, membrane transport, amino acid metabolism, metabolism of cofactors and vitamins, signal transduction, cellular community-prokaryotes, nucleotide metabolism, and drug resistance: antimicrobial.

### 3.9. KEGG Enrichment Analysis

The KEGG enrichment analysis result (Figure 5B) showed that oxidative phosphorylation was the most enriched pathway containing 15 DEGs, followed by the citrate cycle pathway containing 11 DEGs. The other enriched pathways were carbon fixation pathways in ABC transporters, glyoxylate and dicarboxylate metabolism, carbon fixation pathways in prokaryotes, glycine, serine and threonine metabolism, purine metabolism, lipoic acid metabolism, quorum sensing, two-component system, etc.

### 3.10. VFDB Classification Analysis

**Figure 6:**
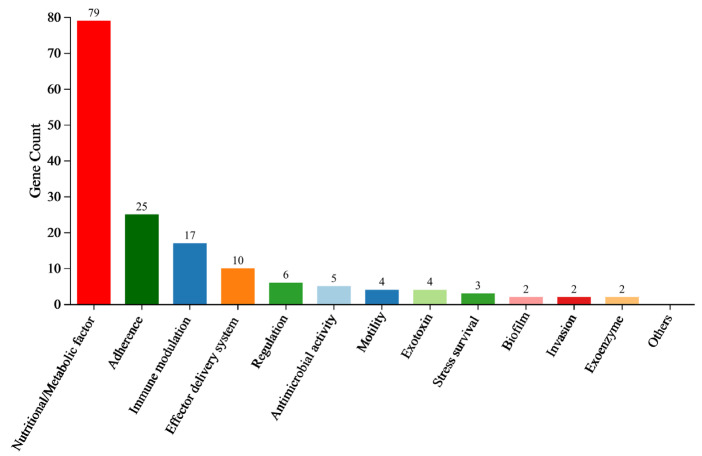
VFDB classification of DEGs between the control and fisetin-treated bacterial cells.

VFDB results showed that 159 DEGs related to the virulence factors were found in *A. hydrophila* XS-91-4-1 after treatment with fisetin, containing nutritional/metabolic factors, adherence, immune modulation, effector delivery systems, regulation, antimicrobial activity, motility, exotoxins, stress survival, invasion, exoenzymes, and biofilm (Figure 6). The representative downregulated genes are listed in Table 3.

## 4. Discussion

Antibiotics in aquatic environments provide selective pressures to bacteria and promote the transfer of antibiotic resistant genes (ARGs) [25]. Studies have demonstrated that *A. hydrophila* strains developed multi-resistance due to the misuse of antibiotics in aquaculture [26,27]. Moreover, antibiotic resistant bacteria (ARB) and ARGs are classified as new classes of environmental pollutants. The ARB and ARGs that exist in aquatic environments not only affect the safety of the ecological environment but also threaten human health [28]. Therefore, novel approaches dealing with antibiotic-resistant bacteria are needed for improving the health of aquatic animals. In recent years, several alternative approaches have been developed to deal with bacterial diseases in aquaculture, such as bacteriophages, short-chain fatty acids, and anti-virulence strategies [29].

Here, the anti-virulence strategy was used to investigate whether fisetin could decrease the pathogenicity of *A. hydrophila* by inhibiting certain virulence factors. Although the MIC of fisetin was 128 μg/mL, we found that fisetin could affect the secretion of aerolysin and biofilm formation at concentrations 64 times lower than the MIC, and a significant protection was observed for fish challenged with *A. hydrophila*. Our previous studies investigated the mechanisms of several natural compounds inhibiting aerolysin production using qRT-PCR [30]. However, qRT-PCR results could only determine the impact of drugs on specific genes, not the global transcription of the bacteria after treatment with drugs. Here, our results showed that fisetin at a concentration of 16 μg/mL did not affect the growth of *A. hydrophila*. Thus, more attention was focused on the inhibitory effect of fisetin against bacterial virulence. The RNA-seq assay was conducted to investigate the global inhibitory effect. Although the results of GO and KEGG enrichment demonstrated that fisetin could affect certain genetic functions and metabolic pathways of *A. hydrophila*, the inhibitory effects of fisetin against virulence factors might be the main approach to decreasing the pathogenicity of *A. hydrophila*. Bacterial oxidative phosphorylation and citrate cycle pathways are not directly involved in the productions of virulence factors but are crucial for energy production and impact the expression of virulence factors. Quorum sensing and two-component system are known that can regulate a number of virulence factors of *A. hydrophila*, such as aerolysin and biofilm formation, as detected in this study. The VFDB classification results showed that a total of 159 DEGs were found, indicating that fisetin could affect the virulence factors of *A. hydrophila*. Toxins and enzymes such as aerolysin, metalloprotease, collagenase, and aerolysin were involved in the pathogenicity of *A. hydrophila*, the expression of which was inhibited according to VFDB classification [31,32]. As is known, adhesion to the host is the first step in the pathogenesis of bacterial pathogens, which plays an essential role in the early infection stage. A total of 25 DEGs were involved in the adhesion of *A. hydrophila*, indicating that fisetin might affect the function of adhesion. Moreover, motility is crucial for adhesion, and our results revealed that fisetin could affect the expression of motility genes of *A. hydrophila*. Bacterial secretory systems are responsible for transporting virulence factors to the medium or host cells, which are essential in the pathogenesis of the bacterium [33]. According to the RNA-seq results, fisetin could suppress seven genes related to the secretory systems. Moreover, VgrG and Hcp, the effectors of the type VI secretion system of *A. hydrophila*, are related to biofilm and virulence factor delivery. Evidence demonstrated that bacterial strains lacking the *vgrG* or *hcp* genes showed a significant reduction in virulence [34]. The findings helped to clarify the mechanism of fisetin inhibiting the virulence of *A. hydrophila*. Several previous studies have demonstrated that the results obtained from RNA-seq technology are the same as the results acquired from qPCR, and validation is not required [35,36]. Thus, we did not validate the RNA-seq results with qPCR.

Fisetin is a well-known anti-virulence agent that can inhibit the expression or activity of virulence factors secreted by several bacterial pathogens and result in a decrease in pathogenicity. The results of Li et al. demonstrated that fisetin could improve the survivability of the murine infection model by inhibiting the type III secretion system regulator HilD of *Salmonella typhimurium* [37]. Zhang et al. showed that fisetin could directly target the activity of suilysin of *Streptococcus suis* serotype 2 and result in a decrease in pathogenicity [38]. Wang et al. found that fisetin could interact with listeriolysin O produced by *Listeria monocytogenes* via binding to loop 2 and 3 of the toxin and lead to a reduction in virulence in vivo [39]. These findings revealed that fisetin is a potent antitoxin agent for seeking drugs against infections. In addition, fisetin is known as an anti-resistance agent by inhibiting the activities of MCR-1 and NDM-1 and can restore the susceptibility of antibiotics [40,41]. However, the inhibitory effect of fisetin against aquatic bacteria has been minimally reported previously. Kang et al. found that fisetin showed antiviral activities against IHNV and VHSV based on a cytopathic effect, indicating that fisetin had the potential as an antiviral drug in dealing with viral infections in aquaculture [42]. Our study determined the mechanism of fisetin using transcriptomic analysis after evaluating the impact of fisetin on hemolysis and biofilm formation of *A. hydrophila*. Animal studies showed that treatment with fisetin could significantly reduce the mortality of fish infected with *A. hydrophila*, suggesting that fisetin is a candidate in dealing with *A. hydrophila* infection in aquaculture. However, the infection model established in the laboratory was completely different from natural infection, and some of the symptoms post infection were not observed in the animal model, which might lead to a change in the treatment effect in aquaculture practice [43]. Therefore, it is necessary to develop animal models based on co-habitation or the immersion challenge, which are much closer to natural infection [43]. Although the findings of the present study indicated that fisetin could be used as an agent controlling *A. hydrophila*-associated infections, there is still a long way for fisetin to be approved as a fishery drug. Moreover, the dose optimization assay is necessary for finding the appropriate dose for practice. The inhibitory ability of fisetin against the pathogenicity of *A. hydrophila* might differ among different bacterial strains because the quantity and expression levels of virulence factors can affect the pathogenicity of *A. hydrophila* [11].

In addition to anti-virulence activity, fisetin is reported to have anti-inflammatory activities in animal models, which might show a synergistic effect in controlling *A. hydrophila* infection [44]. Therefore, to better clarify the mechanism of fisetin as an anti-infective agent, it is necessary to determine the immunomodulatory effect of fisetin on aquatic animals.

## 5. Conclusions

Fisetin with minimal bacteriostatic effects can improve the survivability of channel catfish challenged with *A. hydrophila* under our experimental conditions by suppressing aerolysin and biofilm formation. The feasible mechanism of fisetin was determined using RNA-seq technology. This study offers a potent natural compound in combating bacterial diseases in aquaculture and provides an alternative route in seeking drugs offering less selective pressure than antibiotics.

## Figures and Tables

**Table 1:** The quality control results of RNA-seq data.

Sample Name	Raw Reads	Clean Reads	Clean Bases (bp)	Clean Error Rate (%)	Clean Q20 (%)	Clean Q30 (%)
C1	20,530,174	20,314,872	2,993,496,680	0.0117	98.92	96.59
C2	22,833,722	22,606,096	3,359,219,377	0.0117	98.95	96.61
C3	21,288,572	21,109,952	3,138,989,218	0.0116	98.98	96.70
F1	22,743,550	22,537,754	3,356,574,698	0.0116	98.98	96.71
F2	22,389,376	22,194,338	3,299,969,664	0.0116	99.01	96.78
F3	23,764,044	23,490,540	3,483,066,492	0.0116	98.99	96.73

C, drug-free control group; F, fisetin-treated group.

**Table 2:** Comparison of samples with the genome of *A. hydrophila* ATCC 7966.

Sample Name	Total Reads	Genome Mapped Reads	Genome Mapped Ratio (%)	Unmapped Reads	Unmapped Reads Ratio (%)	Uniq Mapped Reads	Uniq Mapped Reads Ratio (%)
C1	20,314,872	18,679,762	91.95	1,635,110	8.05	18,188,830	89.53
C2	22,606,096	21,479,423	95.02	1,126,673	4.98	21,275,355	94.11
C3	21,109,952	20,056,686	95.01	1,053,266	4.99	19,876,725	94.16
F1	22,537,754	21,468,942	95.26	1,068,812	4.74	21,295,334	94.49
F2	22,194,338	21,203,091	95.53	991,247	4.47	21,065,749	94.91
F3	23,490,540	2,235,0070	95.14	1,140,470	4.86	22,169,827	94.38

C, drug-free control group; F, fisetin-treated group.

**Table 3:** The representative down-regulated genes.

Gene ID	Log_2_ Fold Change	Description
Genes related to exotoxins		
AHA_1194	−1.5085	multidrug resistance efflux pump
AHA_0663	−1.1876	putative transporter
AHA_0438	−1.2324	aerolysin
AHA_0739	−1.1749	putative amidase
Genes related to two-component systems		
AHA_2453	−2.2819	tricarboxylic transport membrane protein TctB
AHA_2454	−2.1815	tricarboxylic transport membrane protein TctA
AHA_2526	−3.3417	hydrogenase small subunit HydA
AHA_2523	−4.0288	hydrogenase-2 large chain HyaB
AHA_3288	−1.0205	two-component system sensor histidine kinase TtrS
Genes related to quorum sensing		
AHA_1305	−1.2950	LasA protease
AHA_2613	−1.1305	oligopeptide transport system substrate OppA
AHA_2517	−1.3323	peptide/nickel transport system ATP DdpD
AHA_2519	−1.4034	peptide/nickel transport system permease protein
AHA_2520	−1.8038	peptide/nickel transport system substrate
Genes related to secretory systems		
AHA_1835	−1.1672	type VI secretion system protein ImpG
AHA_1827	−1.1736	type VI secretion system secreted protein VgrG
AHA_1833	−1.2388	type VI secretion system protein ImpC
AHA_1118	−1.1621	type VI secretion system secreted protein Hcp
AHA_3786	−1.2232	general secretion pathway protein B

## Data Availability

The datasets of RNA-seq can be found in online repositories. The names of the repository/repositories and accession number(s) can be found in the article.

## References

[1] Béné C., Arthur R., Norbury H., Allison E.H., Beveridge M., Bush S., Campling L., Leschen W., Little D., Squires D. (2016). Contribution of Fisheries and Aquaculture to Food Security and Poverty Reduction: Assessing the Current Evidence. World Dev..

[2] Garlock T., Asche F., Anderson J., Ceballos-Concha A., Love D.C., Osmundsen T.C., Pincinato R.B.M. (2022). Aquaculture: The missing contributor in the food security agenda. Glob. Food Secur..

[3] Pridgeon J., Klesius P. (2012). Major bacterial diseases in aquaculture and their vaccine development. CABI Rev..

[4] Qi W., Arthur J.R., Phillips M.J., Subasinghe R.P., Reantaso M.B., MacRae I.H. (2002). Social and economic impacts of aquatic animal health problems in aquaculture in China. Primary Aquatic Animal Health Care in Rural, Small-Scale, Aquaculture Development.

[5] Shirajum Monir M., Yusoff S.M., Mohamad A., Ina-Salwany M.Y. (2020). Vaccination of tilapia against motile *Aeromonas* septicemia: A review. J. Aquat. Anim. Health.

[6] Schwartz K., Borowiak M., Strauch E., Deneke C., Richter M.H., German Aeromonas Study G. (2024). Emerging *Aeromonas* spp. infections in Europe: Characterization of human clinical isolates from German patients. Front. Microbiol..

[7] Farias D.R., Ibarra R., Estevez R.A., Tlusty M.F., Nyberg O., Troell M., Avendano-Herrera R., Norden W. (2024). Towards sustainable antibiotic use in aquaculture and antimicrobial resistance: Participatory experts’ overview and recommendations. Antibiotics.

[8] Lulijwa R., Rupia E.J., Alfaro A.C. (2020). Antibiotic use in aquaculture, policies and regulation, health and environmental risks: A review of the top 15 major producers. Rev. Aquac..

[9] Liu Z., Zhang L., Song Q., Song H., Xu Y., Lu J., Xu Q., Tang Y., Liu Y., Wang G. (2023). Quantitative proteomics reveal the inherent antibiotic resistance mechanism against norfloxacin resistance in *Aeromonas hydrophila*. J. Proteome Res..

[10] Rasmussen-Ivey C.R., Figueras M.J., McGarey D., Liles M.R. (2016). Virulence factors of *Aeromonas hydrophila*: In the wake of reclassification. Front. Microbiol..

[11] Semwal A., Kumar A., Kumar N. (2023). A review on pathogenicity of *Aeromonas hydrophila* and their mitigation through medicinal herbs in aquaculture. Heliyon.

[12] Dawood M.A.O., El Basuini M.F., Yilmaz S., Abdel-Latif H.M.R., Alagawany M., Kari Z.A., Abdul Razab M.K.A., Hamid N.K.A., Moonmanee T., Van Doan H. (2022). Exploring the roles of dietary herbal essential oils in aquaculture: A review. Animals.

[13] Guo H., Chen J., Yuan X., Zhang J., Wang J., Yao J., Ge H. (2023). The combined effect of a novel formula of herbal extracts on bacterial infection and immune response in *Micropterus salmoides*. Front. Microbiol..

[14] Kubina R., Iriti M., Kabala-Dzik A. (2021). Anticancer potential of selected flavonols: Fisetin, kaempferol, and quercetin on head and neck cancers. Nutrients.

[15] Arai Y., Watanabe S., Kimira M., Shimoi K., Mochizuki R., Kinae N. (2000). Dietary intakes of flavonols, flavones and isoflavones by Japanese women and the inverse correlation between quercetin intake and plasma LDL cholesterol concentration. J. Nutr..

[16] Singh S., Singh A.K., Garg G., Rizvi S.I. (2018). Fisetin as a caloric restriction mimetic protects rat brain against aging induced oxidative stress, apoptosis and neurodegeneration. Life Sci..

[17] Wu P.Y., Lyu J.L., Liu Y.J., Chien T.Y., Hsu H.C., Wen K.C., Chiang H.M. (2017). Fisetin regulates Nrf2 expression and the inflammation-related signaling pathway to prevent UVB-induced skin damage in hairless mice. Int. J. Mol. Sci..

[18] Imran M., Saeed F., Gilani S.A., Shariati M.A., Imran A., Afzaal M., Atif M., Tufail T., Anjum F.M. (2021). Fisetin: An anticancer perspective. Food Sci. Nutr..

[19] CLSI (2015). Methods for Antimicrobial Dilution and Disk Susceptibility Testing of Infrequently Isolated or Fastidious Bacteria.

[20] Jayaraman S., Rajendhran N., Kannan M.A., Ramasamy T. (2024). Quercetin disrupts biofilm formation and attenuates virulence of *Aeromonas hydrophila*. Arch. Microbiol..

[21] Mcfarland J. (1907). The nephelometer: An instrument for estimating the number of bacteria in suspensions used for calculating the posonic index and for vaccines. J. Am. Med. Assoc..

[22] Defoirdt T., Bossier P., Sorgeloos P., Verstraete W. (2005). The impact of mutations in the quorum sensing systems of *Aeromonas hydrophila*, *Vibrio anguillarum* and *Vibrio harveyi* on their virulence towards gnotobiotically cultured *Artemia franciscana*. Environ. Microbiol..

[23] Zhang D., Pridgeon J.W., Klesius P.H. (2013). Expression and activity of recombinant proaerolysin derived from *Aeromonas hydrophila* cultured from diseased channel catfish. Vet. Microbiol..

[24] Chakraborty T., Huhle B., Hof H., Bergbauer H., Goebel W. (1987). Marker exchange mutagenesis of the aerolysin determinant in *Aeromonas hydrophila* demonstrates the role of aerolysin in *A. hydrophila*-Associated Systemic Infections. Infect. Immun..

[25] Hossain A., Habibullah-Al-Mamun M., Nagano I., Masunaga S., Kitazawa D., Matsuda H. (2022). Antibiotics, antibiotic-resistant bacteria, and resistance genes in aquaculture: Risks, current concern, and future thinking. Environ. Sci. Pollut. Res. Int..

[26] Peng K., Chen M., Wang Y., Tian Z., Deng L., Li T., Feng Y., Ouyang P., Huang X., Chen D. (2024). Genotype diversity and antibiotic resistance risk in Aeromonas hydrophila in Sichuan, China. Braz. J. Microbiol..

[27] Nhinh D.T., Le D.V., Van K.V., Huong Giang N.T., Dang L.T., Hoai T.D. (2021). Prevalence, virulence gene distribution and alarming the multidrug resistance of *Aeromonas hydrophila* associated with disease outbreaks in freshwater aquaculture. Antibiotics.

[28] Yuan X., Lv Z.Q., Zhang Z.Y., Han Y., Liu Z.Q., Zhang H.J. (2023). A review of antibiotics, antibiotic resistant bacteria, and resistance genes in aquaculture: Occurrence, contamination, and transmission. Toxics.

[29] Defoirdt T., Sorgeloos P., Bossier P. (2011). Alternatives to antibiotics for the control of bacterial disease in aquaculture. Curr. Opin. Microbiol..

[30] Dong J., Zhang L., Liu Y., Zhou S., Yang Y., Xu N., Yang Q., Ai X. (2021). Resveratrol influences the pathogenesis of *Aeromonas hydrophila* by inhibiting production of aerolysin and biofilm. Food Control.

[31] SobhZahedi M., Zamani H., YektaKooshali M.H. (2023). Curcumin aattenuates the expression of metalloprotease (AHA_0978) and serine protease (AHA_3857) genes in *Aeromonas hydrophila*. Galen Med. J..

[32] Duarte A.S., Cavaleiro E., Pereira C., Merino S., Esteves A.C., Duarte E.P., Tomas J.M., Correia A.C. (2015). *Aeromonas piscicola* AH-3 expresses an extracellular collagenase with cytotoxic properties. Lett. Appl. Microbiol..

[33] Fernandez-Bravo A., Figueras M.J. (2020). An update on the genus *Aeromonas*: Taxonomy, epidemiology, and pathogenicity. Microorganisms.

[34] Sha J., Rosenzweig J.A., Kozlova E.V., Wang S., Erova T.E., Kirtley M.L., van Lier C.J., Chopra A.K. (2013). Evaluation of the roles played by Hcp and VgrG type 6 secretion system effectors in *Aeromonas hydrophila* SSU pathogenesis. Microbiology.

[35] Everaert C., Luypaert M., Maag J.L.V., Cheng Q.X., Dinger M.E., Hellemans J., Mestdagh P. (2017). Benchmarking of RNA-sequencing analysis workflows using whole-transcriptome RT-qPCR expression data. Sci. Rep..

[36] Coenye T. (2021). Do results obtained with RNA-sequencing require independent verification?. Biofilm.

[37] Li S., Liu H., Shu J., Li Q., Liu Y., Feng H., Wang J., Deng X., Zhang Y., Guo Z. (2024). Fisetin inhibits *Salmonella* Typhimurium type III secretion system regulator HilD and reduces pathology in vivo. Microbiol. Spectr..

[38] Zhang Y., Zong B., Wang X., Zhu Y., Hu L., Li P., Zhang A., Chen H., Liu M., Tan C. (2018). Fisetin lowers *Streptococcus suis* serotype 2 pathogenicity in mice by inhibiting the hemolytic activity of suilysin. Front. Microbiol..

[39] Wang J., Qiu J., Tan W., Zhang Y., Wang H., Zhou X., Liu S., Feng H., Li W., Niu X. (2015). Fisetin inhibits *Listeria monocytogenes* virulence by interfering with the oligomerization of listeriolysin O. J. Infect. Dis..

[40] Wang N., Sheng Q., Zhu H., Wang J., Qiu J., Cui M., Zhou Y., Deng X., Deng Y., Wang L. (2024). Enhancing the effectiveness of Polymyxin E with a Fisetin Nanoemulsion against a Colistin-resistant *Salmonella typhimurium* infection. Phytomedicine.

[41] Guo Y., Yang Y., Xu X., Li L., Zhou Y., Jia G., Wei L., Yu Q., Wang J. (2022). Metallo-beta-lactamases inhibitor fisetin attenuates meropenem resistance in NDM-1-producing *Escherichia coli*. Eur. J. Med. Chem..

[42] Kang S.Y., Kang J.Y., Oh M.J. (2012). Antiviral activities of flavonoids isolated from the bark of *Rhus verniciflua* stokes against fish pathogenic viruses in vitro. J. Microbiol..

[43] Nordmo R. (1997). Strengths and weaknesses of different challenge methods. Dev. Biol. Stand..

[44] Molagoda I.M.N., Jayasingha J.A.C.C., Choi Y.H., Jayasooriya R.G.P.T., Kang C.-H., Kim G.-Y. (2021). Fisetin inhibits lipopolysaccharide-induced inflammatory response by activating β-catenin, leading to a decrease in endotoxic shock. Sci. Rep..

